# Medicare Insurance Type and Broad Genomic Profiling in Metastatic Cancer

**DOI:** 10.1001/jamanetworkopen.2026.14919

**Published:** 2026-05-27

**Authors:** Ryan D. Chow, John Rothen, Jessica B. Long, Pamela R. Soulos, Xiao Wang, Ronac Mamtani, Shuangge Ma, Natalia Kunst, Michaela A. Dinan, Cary P. Gross

**Affiliations:** 1 Division of Hematology-Oncology, Department of Medicine, Perelman School of Medicine at the University of Pennsylvania, Philadelphia; 2 Yale Cancer Outcomes, Public Policy, and Effectiveness Research Center, Yale Cancer Center, New Haven, Connecticut; 3 Section of Medical Oncology, Department of Internal Medicine, Yale School of Medicine, New Haven, Connecticut; 4 Department of Biostatistics, Yale School of Public Health, New Haven, Connecticut; 5 Centre for Health Economics, University of York, York, United Kingdom; 6 Department of Chronic Disease Epidemiology, Yale School of Public Health, New Haven, Connecticut; 7 Section of General Internal Medicine, Department of Internal Medicine, Yale School of Medicine, New Haven, Connecticut

## Abstract

**Importance:**

Broad genomic profiling (BGP) is recommended for several types of metastatic cancer but remains underused. Over half of Medicare beneficiaries are enrolled in Medicare Advantage (MA), where cost-containment strategies may limit access to BGP. Whether Medicare payer type and geographic region are associated with BGP use is not well established.

**Objectives:**

To evaluate whether BGP use differs by Medicare payer type (MA vs fee-for-service Medicare [FFS]) and to characterize geographic variation in BGP use across hospital referral regions (HRRs).

**Design, Setting, and Participants:**

This nationwide retrospective cohort study used Medicare Chronic Conditions Data Warehouse claims and service records to identify beneficiaries aged 66 years or older with a new diagnosis of metastatic cancer, including bladder, breast, colorectal, endometrial, kidney, lung, melanoma, pancreatic, prostate, or thyroid, from January 1, 2020, to June 30, 2022. Data analysis was conducted from October 2024 to March 2026.

**Exposures:**

Medicare type (FFS vs MA) and HRR.

**Main Outcomes and Measures:**

The primary outcome was receipt of BGP within 2 months before through 6 months after diagnosis. Mixed-effects logistic regression models were used to estimate adjusted odds ratios (AORs) for the association between Medicare type and BGP use, controlling for demographic, clinical, and geographic factors. HRR-level variation was summarized using the median odds ratio (MOR). Subgroup analyses stratified cancers by the strength of guideline recommendations for BGP.

**Results:**

Of 254 720 Medicare beneficiaries with metastatic cancer (median age, 74 years [IQR, 70-79 years]; 141 964 female [55.7%]), 112 637 (44.2%) were enrolled in MA and 142 083 (55.8%) in FFS. Overall, 64 351 (25.3%) received BGP. FFS beneficiaries had higher BGP use than MA beneficiaries (36 633 of 142 083 [25.8%] vs 27 718 of 112 637 [24.6%]; AOR, 1.08 [95% CI, 1.06-1.10]). BGP use was more frequent among FFS vs MA beneficiaries for cancers with equivocal BGP recommendations (AOR, 1.15 [95% CI, 1.11-1.19]) and, to a lesser extent, cancers with explicit recommendations (AOR, 1.04 [95% CI, 1.02-1.07]). Adjusted BGP use varied widely across HRRs (range, 13.8%-35.9%; median, 24.5% [IQR, 21.8%-27.6%]; MOR, 1.28 [95% CI, 1.25-1.31]).

**Conclusions and Relevance:**

In this cohort study of Medicare beneficiaries with metastatic cancer, BGP use differed by Medicare payer type and showed substantial regional variation. These findings highlight opportunities to improve guideline-concordant molecular testing.

## Key Points

**Question:**

Is the use of broad genomic profiling (BGP) associated with Medicare payer type or hospital referral region (HRR) among Medicare beneficiaries with metastatic cancer?

**Findings:**

In this nationwide cohort study of 254 720 Medicare beneficiaries with newly diagnosed metastatic cancer, BGP use was significantly higher among fee-for-service Medicare than Medicare Advantage enrollees, including for cancers with explicit guideline recommendations for BGP. BGP use also varied markedly across HRRs.

**Meaning:**

Findings suggest that BGP use differed by Medicare payer type and showed substantial regional variation, underscoring the need for policies that support guideline-concordant molecular testing for patients with metastatic cancer.

## Teaser

This cohort study of more than 250 000 Medicare beneficiaries evaluates whether broad genomic profiling use differs by Medicare Advantage vs fee-for-service Medicare and characterizes geographic variation in broad genomic profiling use across hospital referral regions.

## Introduction

With the growing armamentarium of molecularly targeted cancer therapies,^1^ current national guidelines explicitly recommend broad genomic profiling (BGP) for several types of metastatic cancer.^2^ Accordingly, Medicare issued a national coverage determination in 2018 for certain types of BGP testing for patients with metastatic cancer.^3^ Although BGP use has increased over time, it remains underused.^4,5,6,7,8,9,10^ A deeper understanding of the barriers to BGP use is key to ensuring high-quality cancer care. In addition to patient-related factors,^4^ these barriers may arise from broader features of the health systems in which patients are treated. In particular, payer policies (such as differences between Medicare Advantage [MA] and fee-for-service [FFS] Medicare plans) and geographic variation in the structure or culture of local health systems may all influence access to BGP.

MA plans now account for most Medicare enrollment, supplanting traditional FFS Medicare.^11^ In contrast with FFS Medicare plans, MA plans are administered by corporate entities that receive federal dollars to pay for Medicare services. By virtue of their capitated payment model, MA plans are incentivized to limit health care spending. Accordingly, MA coverage has been associated with greater use of preventive care, along with fewer hospital admissions and emergency department visits.^12^ In addition, MA beneficiaries undergoing chemotherapy have been shown to incur lower Medicare Part B chemotherapy-related costs than FFS Medicare beneficiaries, without compromising select quality outcomes.^13^ MA plans are also associated with decreased use of low-value care, including unnecessary cancer screening^14,15^ and treatments.^16^

However, the cost-controlling restrictions inherent to MA plans may impose barriers to receipt of high-quality cancer care. Among Medicare beneficiaries in California, MA coverage was associated with delayed treatment initiation and reduced survival among patients with metastatic lung cancer.^17^ Cost containment strategies also vary among MA plans, which is associated with heterogeneity in the quality of care, prior authorization requirements, and restrictiveness of clinician networks^18,19,20,21,22,23,24,25^; these network restrictions can impede access to high-volume cancer centers.^26,27^ Given that MA plans are incentivized to limit overuse of unnecessary care, it is imperative to examine whether they are simultaneously associated with the underuse of guideline-recommended interventions, such as BGP. Conversely, FFS Medicare programs may be more vulnerable to overuse of BGP in scenarios where testing has limited clinical value. Accordingly, evaluating BGP use for cancers for which testing is recommended by national guidelines, as well as for cancers for which it is not, is essential for distinguishing appropriate testing from overuse or underuse.

In addition to payer type, cancer care also varies by geographic region, with differences in the structure and culture of local health systems. Cancer incidence and mortality rates differ across states,^28,29^ reflecting variability in care delivery and social determinants of health.^30,31^ For instance, 24% to 48% of the observed variation in breast cancer treatment decisions is attributable to geographic region, compared with the 1% to 4% variation from patient-level factors.^32^ Similarly, the nationwide adoption of new genomic tests for patients with prostate cancer has differed considerably across hospital referral regions (HRRs).^33^ To date, little is known about whether BGP use varies across HRRs. A deeper understanding of geographic variation in BGP use is key for ensuring equitable access to precision oncology.

In this population-level analysis, we examined BGP use among Medicare beneficiaries with newly diagnosed metastatic cancer from 2020 to 2022. We evaluated whether BGP use differed by Medicare payer type (MA vs FFS) and characterized variation across HRRs, further stratifying by cancer type and the strength of contemporaneous guideline recommendations for BGP. Our objective was to identify payer- and region-level factors associated with access to guideline-concordant genomic testing in modern oncology care.

## Methods

### Data Source and Study Population

The Yale University institutional review board determined that this study was exempt from review and waived informed consent because beneficiaries were deidentified. We followed the Strengthening the Reporting of Observational Studies in Epidemiology (STROBE) reporting guideline and checklist for cohort studies. We used data from the Chronic Conditions Data Warehouse, a Medicare beneficiary, claims, and assessment database maintained by the Centers for Medicare & Medicaid Services (CMS). The Medicare data are split into 2 sections: 1 for FFS Medicare and 1 for MA data submitted to the CMS. To ensure comparability across our 2 samples, we omitted diagnosis codes derived from medical record reviews, as they were available only in MA service records.

Our study population consisted of Medicare patients aged 66 years or older with an *International Statistical Classification of Diseases and Related Health Problems, Tenth Revision* (*ICD-10*) diagnosis code corresponding to 1 of the 10 most common solid cancers in the Surveillance, Epidemiology, and End Results Cancer Statistics Review^34^ (bladder, breast, colorectal, endometrial, kidney, lung, melanoma, pancreatic, prostate, and thyroid cancer), billed between January 1, 2020, and June 30, 2022. We did not further stratify patients by specific histopathologic cancer types (eg, adenocarcinoma vs squamous cell carcinoma) due to data limitations. We restricted the sample to patients with metastatic cancer based on the presence of a secondary malignant neoplasm *ICD-10* code (C77, C78, or C79) in the 2 months before through 6 months after their initial cancer diagnosis. Patients with a solid tumor cancer diagnosis code in the year prior to their index date were excluded, as we aimed to focus on patients with incident disease. To ensure specificity, we required the diagnosis code to appear on 1 or more inpatient claim dates, or on 2 or more outpatient or physician claim dates within 2 months; for the latter, we set the diagnosis date as the date of the earliest qualifying claim. Patients without continuous Medicare coverage (Parts A and B for FFS and Part C for MA) for the 12 months prior through 6 months after the diagnosis date were excluded; thus, patients with mixed FFS and MA coverage within the study eligibility window were excluded. Additional details of the cohort are described in the eMethods and eTable 1 in Supplement 1.

### Variable Construction

We assessed BGP use in the 2 months before through 6 months after initial cancer diagnosis. We defined BGP as the sequencing of 10 or more genes in a single day. To identify these BGP events in claims or service records, we integrated 3 complementary approaches^4,7^: (1) established codes: specific *Current Procedural Terminology* (*CPT*) and Healthcare Common Procedure Coding System (HCPCS) codes corresponding to known BGP tests; (2) stacked codes: a collection of individual gene-sequencing *CPT* and HCPCS codes occurring on the same date (a common approach for billing BGP, particularly when established codes have not yet been standardized); and (3) generic codes: use of a code that is not restricted explicitly to BGP tests but is known to be used for billing BGP. Details of included codes are in eTable 2 in Supplement 1.

We stratified age at diagnosis into 4 categories (66-70, 71-75, 76-80, and ≥81 years). We determined race and ethnicity, sex, and Medicaid dual-eligibility status from member information in the Master Beneficiary Summary File. Race and ethnicity were included as covariates because of their known associations with healthcare access and utilization, as well as their potential to confound the association between Medicare type and receipt of BGP. We used the Research Triangle Institute^35^ codes for race and ethnicity, retaining their categorization: American Indian or Alaska Native, Asian or Pacific Islander, Hispanic, non-Hispanic Black, non-Hispanic White, other (individuals who were not placed into any other category per the Research Triangle Institute algorithm; this includes multiracial individuals or people who identify with a race or ethnicity that is not represented in the specified categories), or unknown. Patients were classified as Medicaid dual eligible if they were eligible for both Medicare and Medicaid at any point during the 12 months leading up to and including the month of their diagnosis. We calculated the Elixhauser Comorbidity Index^36^ using data from the year before cancer diagnosis and split into 3 categories (0, 1-2, and ≥3 conditions). The frailty score was calculated using the year of data prior to diagnosis and then split into a categorical variable where patients with a Kim Computed Frailty Index^37^ of 0.2 or more were identified as frail. We used the FIPS (Federal Information Processing Standards) code in the patient’s cancer diagnosis month to assign census region and metropolitan residence status by linking 2023 Rural-Urban Continuum Codes.^38^ We derived the Social Deprivation Index (SDI)^39^ using patient zip codes in the year of diagnosis, then split these into quartiles as a measure of area-level socioeconomic status. We assigned patients to HRRs^40^ based on their zip code in the year of diagnosis; all HRRs had 100 or more eligible patients and thus were included for analysis.

For each cancer type, we evaluated contemporaneous National Comprehensive Cancer Network (NCCN) guideline recommendations for BGP use among patients with metastatic disease. We categorized cancer types into 3 groups based on the strength of BGP guideline recommendations at the beginning of the assessed time period (2020): explicitly recommended (lung, pancreas, melanoma, and breast cancer); equivocally recommended (colorectal, endometrial, bladder, thyroid, and prostate cancer); or not routinely recommended (kidney cancer), as previously described.^4^ Although subsequent NCCN guidelines have expanded BGP recommendations to reflect novel targeted therapies approved after 2020, we opted to focus on contemporaneous guidelines to best reflect the therapeutic landscape at the time of diagnosis.

### Statistical Analysis

Statistical analysis was conducted from October 2024 to March 2026. We calculated summary statistics to visualize temporal trends in BGP use, stratified by Medicare type. We constructed mixed-effects logistic regression models to identify factors associated with BGP use. Our primary independent variable was Medicare type (FFS vs MA). We controlled for time of diagnosis, Medicaid dual eligibility, age group, sex, race and ethnicity, census region, metropolitan status, comorbidity group, frailty status, primary cancer site, and SDI. Subgroup analyses focused on different groups of cancers, categorized by the strength of guideline recommendations for BGP. We also conducted lung cancer–specific analyses, as BGP use was highest and had the longest-standing recommendation in this subgroup. We conducted additional analyses with an interaction term (diagnosis time × Medicare type) to assess whether the association of BGP use with Medicare type (FFS vs MA) varied over time. To account for regional variation in BGP use, we included HRR as a random effect. Significance of variables was estimated using the Wald *t* test and the *F* test. Tests were 2-sided, and *P* < .05 and 95% CIs not including 1.00 were considered statistically significant.

We analyzed HRR-level variation in BGP use by calculating the adjusted BGP rate for each HRR, using the mixed-effects model with a random HRR-level effect and accounting for diagnosis time, primary cancer site, and Medicare type. To compare HRR-level BGP use in MA vs FFS, we filtered for HRRs with 100 or more patients in both cohorts and adjusted for diagnosis time and primary cancer site. We also assessed the correlation between unadjusted BGP rates and MA participation rates in 2021 at the HRR level. To describe variability in BGP use across HRRs, we calculated the median odds ratio (MOR) in the fully adjusted model, which describes the median increase in odds of BGP use when moving from a lower-use HRR to a higher-use HRR.^41^ The MOR 95% CIs were calculated by the transforming variance method.^42^ All analyses were performed using SAS Enterprise Guide, version 8.5 (SAS Institute Inc).

## Results

### BGP Use Across Cancer Types

**Figure 1.**
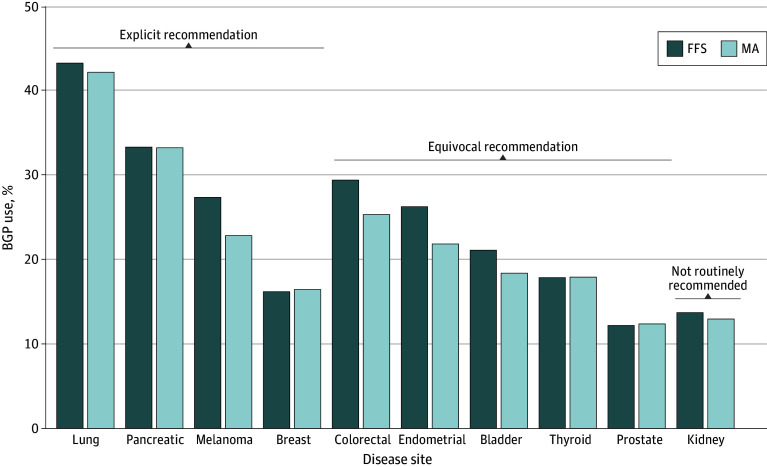
Bar Graph Showing Broad Genomic Profiling (BGP) Use Across Disease Sites Use of BGP among patients with metastatic cancer, stratified by primary cancer site and Medicare type. FFS indicates fee-for-service Medicare; MA, Medicare Advantage.

A total of 254 720 patients with metastatic cancer met the inclusion criteria (median age, 74 years [IQR, 70-79 years]; 141 964 female [55.7%] and 112 756 male [44.3%]; 862 American Indian or Alaska Native [0.3%], 6535 Asian or Pacific Islander [2.6%], 16 065 Hispanic [6.3%], 24 036 non-Hispanic Black [9.4%], 200 608 non-Hispanic White [78.8%], 2222 other race or ethnicity [0.9%], and 4392 unknown race or ethnicity [1.7%]), of whom 64 351 patients (25.3%) received BGP (Table). There were 142 083 patients (55.8%) enrolled under FFS Medicare and 112 637 patients (44.2%) under MA. In aggregate, 36 633 of 142 083 FFS beneficiaries (25.8%) underwent BGP testing from 2020 to 2022 compared with 27 718 of 112 637 MA beneficiaries (24.6%). BGP use was highest among patients with lung, pancreatic, and colorectal cancers (Figure 1).^4^ Even for cancer types with explicit recommendations for BGP use (lung, pancreatic, melanoma, and breast), fewer than half of eligible patients received BGP.

**Table.** Baseline Characteristics of the Study Cohort

Characteristic	No. (%)	
	Fee-for-service Medicare (n = 142 083)	Medicare Advantage (n = 112 637)
BGP use		
No BGP	105 450 (74.2)	84 919 (75.4)
BGP	36 633 (25.8)	27 718 (24.6)
Sex		
Male	63 331 (44.6)	49 425 (43.9)
Female	78 752 (55.4)	63 212 (56.1)
Cancer site		
Bladder	4501 (3.2)	3349 (3.0)
Breast	34 036 (24.0)	27 718 (24.6)
Colorectal	19 927 (14.0)	16 484 (14.6)
Endometrial	6554 (4.6)	5228 (4.6)
Kidney	4842 (3.4)	3495 (3.1)
Lung	33 031 (23.3)	27 037 (24.0)
Melanoma	7110 (5.0)	4629 (4.1)
Pancreatic	7761 (5.5)	5518 (4.9)
Prostate	21 993 (15.5)	17 461 (15.5)
Thyroid	2328 (1.6)	1718 (1.5)
Race and ethnicity		
American Indian or Alaska Native	671 (0.5)	191 (0.2)
Asian or Pacific Islander	3452 (2.4)	3083 (2.7)
Hispanic	5066 (3.6)	10 999 (9.8)
Non-Hispanic Black	9088 (6.4)	14 948 (13.3)
Non-Hispanic White	119 798 (84.3)	80 810 (71.7)
Other^a^	1251 (0.9)	971 (0.9)
Unknown	2757 (1.9)	1635 (1.5)
Age group, y		
66-70	39 859 (28.1)	32 584 (28.9)
71-75	40 500 (28.5)	32 992 (29.3)
76-80	30 281 (21.3)	24 963 (22.2)
≥81	31 443 (22.1)	22 098 (19.6)
Elixhauser Comorbidity Index		
0	46 467 (32.7)	33 426 (29.7)
1-2	53 414 (37.6)	42 298 (37.6)
≥3	42 202 (29.7)	36 913 (32.8)
Claims-based frailty index		
Not frail	107 608 (75.7)	85 127 (75.6)
Frail	34 475 (24.3)	27 510 (24.4)
SDI quartile		
1 (Least deprived)	32 102 (22.6)	24 934 (22.1)
2	33 872 (23.8)	27 860 (24.7)
3	35 976 (25.3)	26 908 (23.9)
4 (Most deprived)	37 730 (26.6)	29 675 (26.4)
Missing	2403 (1.7)	3260 (2.9)
Metropolitan status		
Metropolitan	11 0697 (77.9)	93 826 (83.3)
Nonmetropolitan	29 804 (21.0)	16 315 (14.5)
Missing	1582 (1.1)	2496 (2.2)
Medicaid dual eligibility		
No	126 920 (89.3)	90 871 (80.7)
Yes	15 163 (10.7)	21 766 (19.3)
Census region		
Northeast	26 106 (18.4)	20 703 (18.4)
Midwest	32 888 (23.2)	28 265 (25.1)
South	54 077 (38.1)	43 098 (38.3)
West	28 693 (20.2)	17 840 (15.8)
Missing	319 (0.2)	2731 (2.4)

Abbreviations: BGP, broad genomic profiling; SDI, Social Deprivation Index.

^a^ Refers to individuals who are not placed into any other category per the Research Triangle Institute algorithm; this includes multiracial individuals or people who identify with a race or ethnicity that is not represented in the specified categories.

### Association of Medicare Type With BGP Use

**Figure 2.**
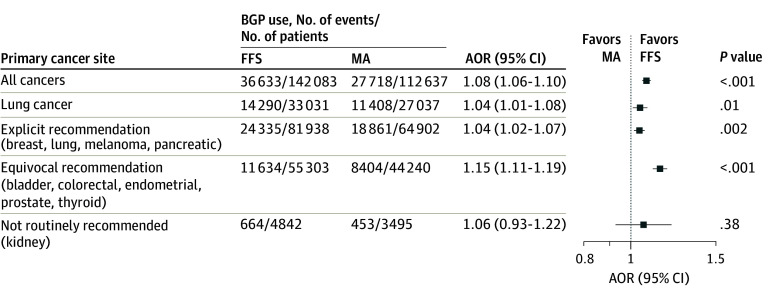
Forest Plot Showing Association of Medicare Type With Broad Genomic Profiling (BGP) Use Among Patients With Metastatic Cancer AOR indicates adjusted odds ratio; FFS, fee-for-service Medicare; and MA, Medicare Advantage.

FFS beneficiaries were more likely to receive BGP testing than MA beneficiaries (36 633 of 142 083 [25.8%] vs 27 718 of 112 637 [24.6%]; adjusted odds ratio [AOR], 1.08 [95% CI, 1.06-1.10]) (Figure 2), with adjusted BGP use of 23.0% (95% CI, 22.4%-23.6%) among FFS beneficiaries compared with 21.7% (95% CI, 21.1%-22.3%) among MA beneficiaries over the study period. Other factors associated with BGP use included age, comorbidity, frailty, SDI, and Medicaid dual eligibility (eTable 3 in Supplement 1). We conducted subgroup analyses stratifying cancer sites by the strength of BGP guideline recommendations (eTables 4-7 in Supplement 1). Compared with MA, FFS coverage was associated with higher BGP use for lung cancer (AOR, 1.04 [95% CI, 1.01-1.08]) and cancers with explicit (AOR, 1.04 [95% CI, 1.02-1.07]) or equivocal (AOR, 1.15 [95% CI, 1.11-1.19]) recommendations for BGP, but not for kidney cancer (AOR, 1.06 [95% CI, 0.93-1.22]), for which BGP is not recommended (Figure 2).

### Temporal Trends in BGP Use by Medicare Type

**Figure 3.**
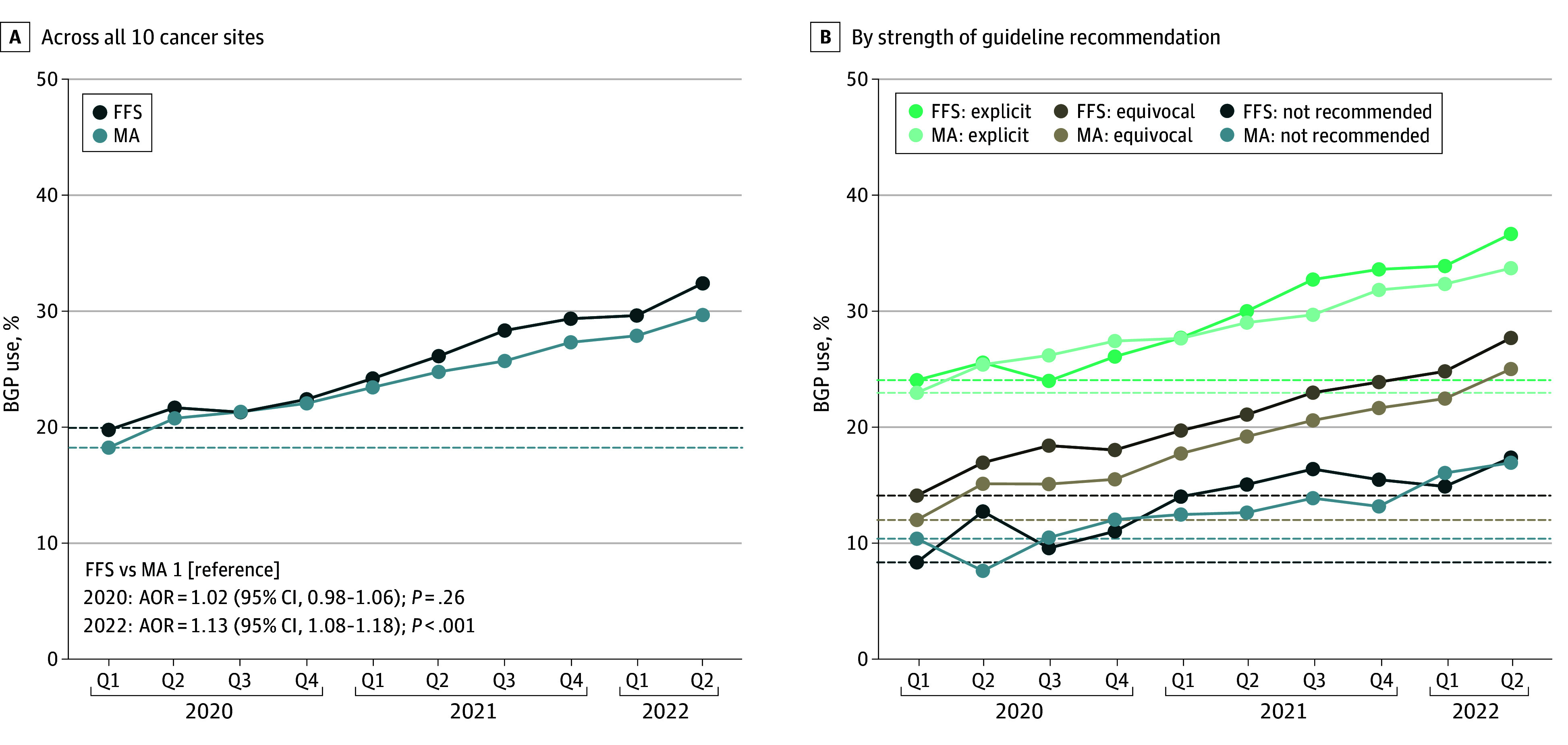
Line Graphs Showing Trends in Broad Genomic Profiling (BGP) Use From 2020 to 2022 BGP use from 2020 to 2022 among Medicare Advantage (MA) or fee-for-service Medicare (FFS) beneficiaries who received a diagnosis of a metastatic cancer, aggregated across all 10 cancer sites (A) or stratified by the strength of BGP guideline recommendations (B). Q indicates quarter.

From quarter 1 of 2020 to quarter 2 of 2022, BGP use increased from 19.7% (2628 of 13 312) to 32.4% (4591 of 14 172) for FFS (64.1% relative increase) compared with an increase from 18.2% (1691 of 9286) to 29.7% (3900 of 13 149) for MA (62.9% relative increase) (Figure 3A). In adjusted analyses, BGP use throughout 2020 was comparable between FFS and MA beneficiaries (AOR, 1.02 [95% CI, 0.98-1.06]). By 2022, however, FFS beneficiaries were significantly more likely to receive BGP compared with MA beneficiaries (AOR, 1.13 [95% CI, 1.08-1.18]). Consistent with a faster rate of BGP adoption among FFS beneficiaries, the diagnosis year and quarter × Medicare type interaction on BGP use was statistically significant (*P* = .004). Across cancer sites with varying strengths of BGP guideline recommendations, BGP use similarly increased from 2020 to 2022 (Figure 3B). Among cancers for which BGP is explicitly recommended, BGP remained at or below 50% throughout the study period (eFigure in Supplement 1).

### Regional Variation in BGP Use

**Figure 4.**
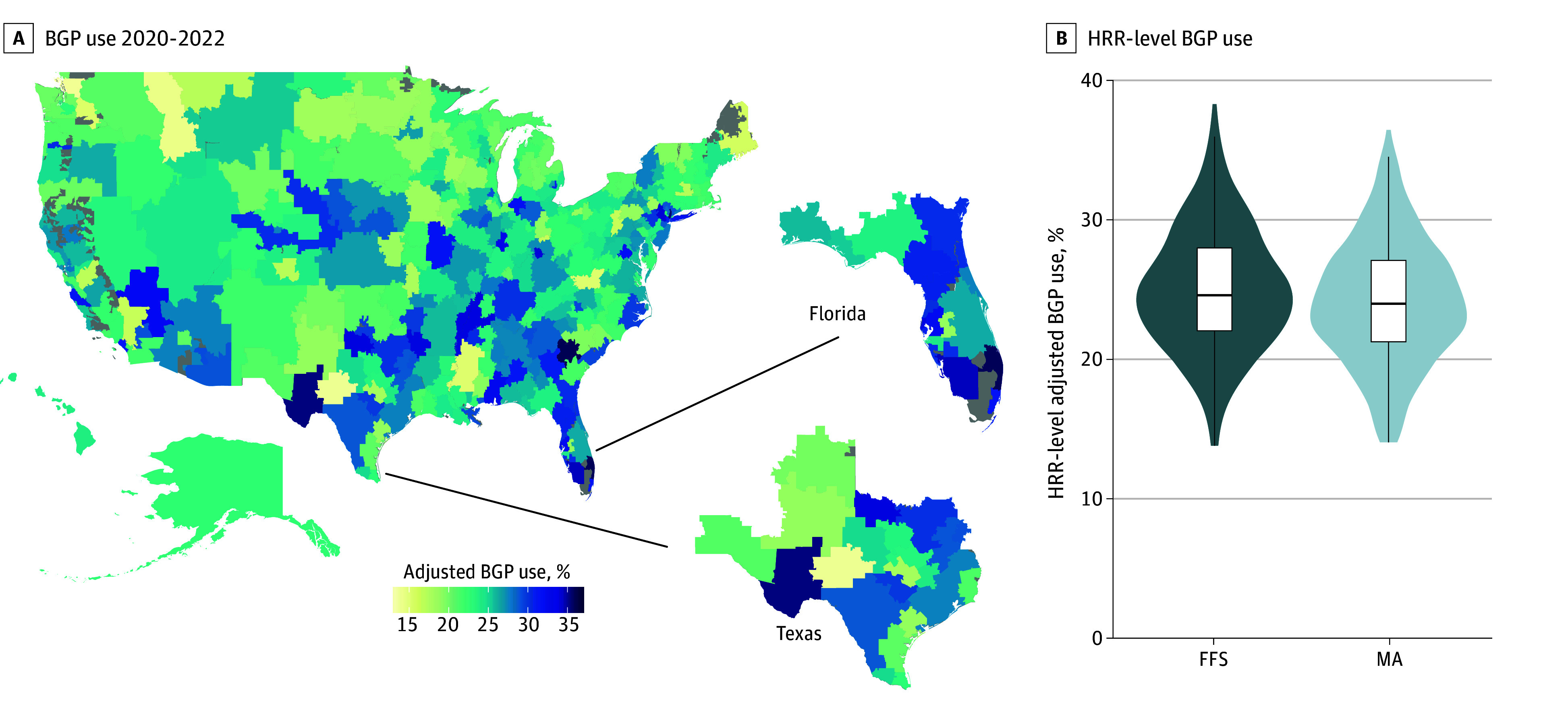
Map and Violin Plot of Variation in Broad Genomic Profiling (BGP) Use Across Hospital Referral Regions (HRRs) A, Map of broad genomic profiling use from 2020 to 2022 among patients with metastatic cancer, grouped by HRRs and adjusted for primary cancer site, Medicare type, and diagnosis time. B, Violin plot showing HRR-level BGP use, adjusted for primary cancer site and diagnosis time, stratified by Medicare type. Box plots show the median and IQR, with whiskers extending to the most extreme values within 1.5 × IQR. FFS indicates fee-for-service Medicare; MA, Medicare Advantage.

After adjustment for diagnosis time, primary cancer site, and Medicare type, BGP use exhibited considerable variation across HRRs, ranging from 13.8% to 35.9% (median, 24.5% [IQR, 21.8%-27.6%]) (Figure 4A). Such variations were even observed among HRRs within individual states. The HRR with the second-lowest adjusted BGP use (San Angelo, Texas; 14.0%) was directly contiguous with the HRR with the fourth-highest BGP use nationwide (Odessa, Texas; 35.2%). Unadjusted BGP use was not correlated with 2021 regional-level MA participation (Pearson *R* = 0.01 [95% CI, −0.10 to 0.13]). We further assessed HRR-level BGP use stratified by Medicare type, adjusting for diagnosis time and primary cancer site (Figure 4B). HRR-level adjusted BGP use ranged from 14.2% to 34.6% in MA (median, 24.0% [IQR, 21.4%-27.3%]) and from 13.9% to 38.3% in FFS (median, 24.8% [IQR, 22.2%-28.2%]).

As a quantitative measure of HRR-level variability in BGP use, the MOR was 1.28 (95% CI, 1.25-1.31) in the fully adjusted model, indicating that for 2 patients matched by clinical, socioeconomic, and demographic characteristics, the odds of BGP receipt were 28.0% higher in an HRR with high BGP use compared with an HRR with low BGP use. Geographic variability in BGP use was comparable between MA (MOR, 1.31 [95% CI, 1.27-1.35]) and FFS (MOR, 1.28 [95% CI, 1.24-1.31]). Similar geographic variation was observed in subgroup analyses of cancers for which BGP is explicitly recommended (MOR, 1.31 [95% CI, 1.28-1.35]), including lung cancer (MOR, 1.38 [95% CI, 1.33-1.43]), as well as cancers with equivocal BGP recommendations (MOR, 1.27 [95% CI, 1.23-1.31]) and those for which BGP is not recommended (MOR, 1.40 [95% CI, 1.23-1.54]).

## Discussion

In this nationwide cohort of Medicare beneficiaries with newly diagnosed metastatic cancer from 2020 to 2022, BGP use varied substantially by Medicare type and geographic region. FFS Medicare beneficiaries were more likely to undergo BGP than beneficiaries enrolled in MA, and this difference widened over time. In parallel, BGP use varied widely across HRRs, including for cancers for which BGP is explicitly recommended.

To our knowledge, this study is the first to comprehensively evaluate payer-related variation in BGP use within a contemporary Medicare population. Our observation that BGP was more rapidly adopted among FFS beneficiaries suggests that cost containment strategies within MA plans may be associated with the uptake of guideline-recommended molecular testing. The association of Medicare payer type with BGP use was stronger for cancers with equivocal guideline recommendations for BGP compared with cancers for which BGP is explicitly recommended. This finding suggests that payer-level utilization management may have a greater influence when clinical guidance is less definitive.

In addition to payer-related differences, we observed marked geographic variation in BGP use across HRRs, underscoring the association of local practice patterns, health system infrastructure, and payer networks in shaping access to genomic testing, as previously demonstrated with other aspects of cancer care.^30,31,32,33,43^ In some cases, neighboring HRRs within the same state showed over 2-fold differences in adjusted BGP use.

More generally, BGP was underused relative to clinical guideline recommendations; for cancers for which BGP is explicitly recommended, fewer than half of patients underwent testing. Beyond payer type and geographic region, other potential barriers to guideline-recommended testing include limited biopsy tissue quantity or quality,^44^ anticipated delays in care while awaiting BGP results,^45^ inability to reliably obtain targeted therapies for underinsured patients,^46^ and reimbursement or claim denial concerns.^47,48^ Identifying and addressing such barriers will be crucial for ensuring high-quality cancer care in the era of molecular medicine.

### Limitations

This study has several limitations. First, we relied on billing codes to determine BGP use, potentially leading to both underascertainment (missed BGP testing) and overascertainment (incorrect identification of a test as BGP). The latter risk is especially pertinent, as we could not apply our previous cost threshold for generic codes^4^ due to the absence of cost information for MA patients in the dataset. Second, we were not able to stratify patients by specific histopathologic cancer types, which would inform the utility of BGP testing in clinical practice. For instance, BGP is critical for guiding the treatment of non–small cell lung cancer but has limited utility for small cell lung cancer; these subtypes of lung cancer were not distinguishable in our analysis. Third, despite adjustment for a comprehensive array of demographic, clinical, and geographic factors, we cannot rule out residual confounding in our models. Fourth, the study period overlapped with the COVID-19 pandemic, which may have affected access to cancer care, potentially influencing patterns of BGP use. Fifth, our results may not be generalizable to younger, commercially insured populations, although many of the factors associated with BGP use in the present cohort study were similarly identified in a recent study of privately insured patients.^4^

## Conclusions

In this cohort study of Medicare beneficiaries with a new diagnosis of metastatic cancer from 2020 to 2022, BGP use varied by payer type and geographic region. Among patients with metastatic cancers for which BGP is explicitly recommended, fewer than half received testing. Our findings have important implications for health policy and cancer care delivery. Given the comparatively lower rates of BGP use among MA beneficiaries, policymakers and regulators may need to consider whether MA quality metrics sufficiently account for guideline-recommended genomic testing. Incorporating measures of appropriate BGP use into value-based payment models or quality reporting programs could help reduce underuse among eligible patients. Beyond payer policy, the remarkable regional variation in BGP also underscores the need for interventions to ensure equitable access to BGP nationwide, such as regionally targeted educational initiatives to improve awareness of guideline recommendations for BGP.

## Notes

Supplement 1. **eFigure. **Temporal Trends of BGP Use in Cancers With Explicit BGP Recommendations **eTable 1. **Cohort Selection Strategy **eTable 2.** BGP Codes Used for Analysis **eTable 3.** Regression Results From All Ten Cancer Sites **eTable 4.** Regression Results From Lung Cancers Only **eTable 5.** Regression Results From Cancers With Explicit BGP Recommendation **eTable 6.** Regression Results From Cancers With Equivocal BGP Recommendation **eTable 7.** Regression Results From Cancers for Which BGP Is Not Routinely Recommended
eMethods.

Supplement 2.
Data Sharing Statement
