# Supplementary material for: Medicare Insurance Type and Broad Genomic Profiling in Metastatic Cancer
Source: JAMA Netw Open. 2026 May 27;9(5):e2614919. doi: 10.1001/jamanetworkopen.2026.14919 (PMC13216988; doi:10.1001/jamanetworkopen.2026.14919)
Supplement: Supplement 1. — eFigure. Temporal Trends of BGP Use in Cancers With Explicit BGP Recommendations eTable 1. Cohort Selection Strategy eTable 2. BGP Codes Used for Analysis eTable 3. Regression Results From All Ten Cancer Sites eTable 4. Regression Results From Lung Cancers Only eTable 5. Regression Results From Cancers With Explicit BGP Recommendation eTable 6. Regression Results From Cancers With Equivocal BGP Recommendation eTable 7. Regression Results From Cancers for Which BGP Is Not Routinely Recommended eMethods. [file jamanetwopen-e2614919-s001.pdf]

Supplementary Online Content

Chow RD, Rothen J, Long JB, et al. Medicare insurance type and broad genomic profiling in metastatic cancer. *JAMA Netw Open.* 2026;9(5):e2614919. doi:10.1001/jamanetworkopen.2026.14919

eFigure. Temporal Trends of BGP Use in Cancers With Explicit BGP Recommendations
eTable 1. Cohort Selection Strategy
eTable 2. BGP Codes Used for Analysis
eTable 3. Regression Results From All Ten Cancer Sites
eTable 4. Regression Results From Lung Cancers Only
eTable 5. Regression Results From Cancers With Explicit BGP Recommendation
eTable 6. Regression Results From Cancers With Equivocal BGP Recommendation
eTable 7. Regression Results From Cancers for Which BGP Is Not Routinely Recommended
eMethods.

This supplementary material has been provided by the authors to give readers additional information about their work.

© 2026 Chow RD et al. *JAMA Network Open.*

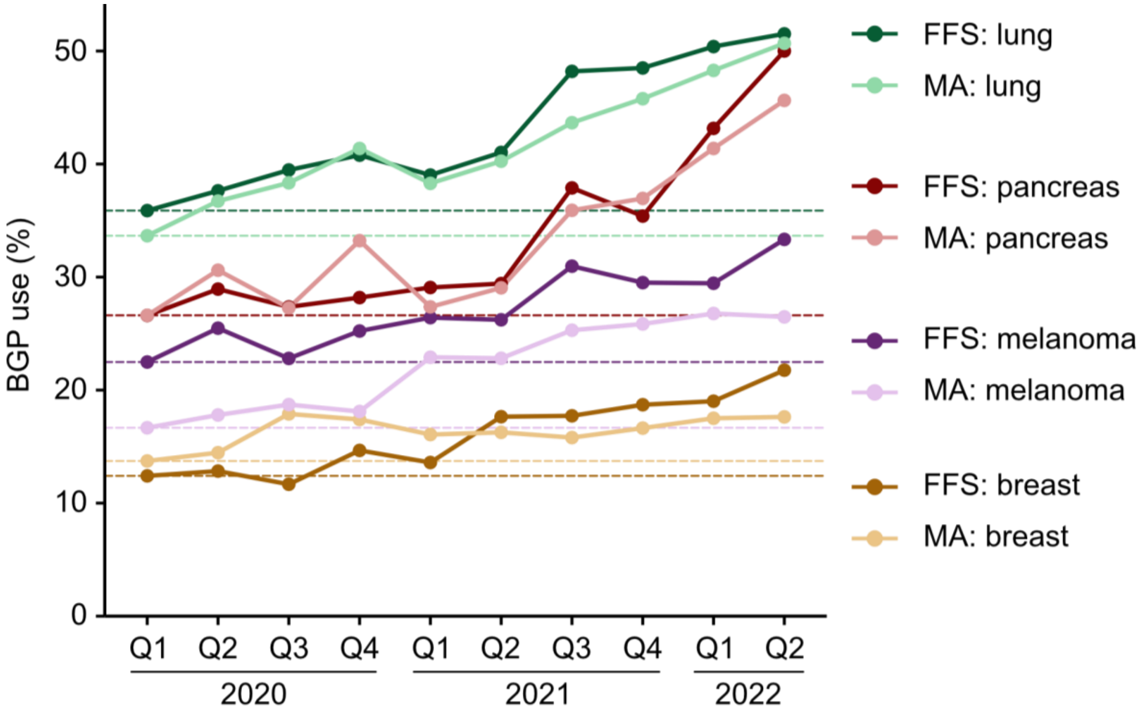

eFigure. Temporal Trends of BGP Use in Cancers With Explicit BGP Recommendations
BGP use from 2020-2022 in MA or FFS beneficiaries diagnosed with metastatic cancer of the lung, pancreas, melanoma, or breast.

© 2026 Chow RD et al. *JAMA Network Open.*

eTable 1. Cohort Selection Strategy

FFS (N)	MA (N)	Description
2684565	1513630	Patients with a diagnosis date after 2020. Note, FFS has a wider range of available claims, and are restricted further down.
2664005	1513421	Patients have no other study-cancer in the 12 months prior to diagnosis
2657294	1510730	Patients who do not have multiple cancers diagnosed on the same day
1525774	910068	Patients have relevant cancer diagnosis appear on multiple claims in 60 days or on an inpatient claim
1366734	795907	Patients aged 66 or greater at diagnosis
929952	562347	Patients have continuous enrollment in AB. Patients also must have all-HMO coverage, or no HMO coverage for MA/FFS respectively
669181	.	Patient diagnosed before the end of June 2022 (matching FFS/MA timeframe)
.	525845	Patients in with outlier MA parent plan
669164	525634	If a patient has two qualifying eligibility episodes in our data, only take the earliest (de-duplicating)
<142094	<112648	Metastatic diagnosis within the 12m prior to 6months after diagnosis (actual value not shown to protect patient privacy by calculable value <11)
142083	112637	Sex information not missing

eTable 2. BGP Codes Used for Analysis

Definite Codes

Code	# of genes	Name
81455	51+	Generic Genomic sequence analysis (solid or hematolymphoid)
81456	51+	Solid/hematolympoid neoplasm analysis; 51+ genes DNA/RNA
0013U	whole exome	Whole genome sequencing (code DELETED 10/1/22)
0019U	~193 proteins	Oncotarget Oncotreat
0022U	23 genes	Oncomine dx target test
0036U	whole exome	Exact-1 whole exome test
0037U	324	F1CDx
0048U	468	MSK-IMPACT
0179U	23	Resolution ctDx Lung
0211U	whole exome	MI Cancer Seek
0239U	311+	F1CDx - Liquid
0242U	55-74	Guardant360 liquid biopsy
0244U	257	Oncotype MAP PanCancer Tissue Test
0250U	505	PGDx elioTM
0326U	83+	Guardant360 ctDNA
0329U	whole exome	Oncomap ExTra
0334U	84+	Guardant360 tissuenext
0409U	80 DNA, 36 RNA	Liquidhallmark

Generic Codes

Code	# of genes	Name
81445	5 to 50	generic solid-organ neoplasm analysis; 5-50 genes DNA/RNA
81479	unspecified	unlisted molecular pathology procedure. NOTE: this code can instead count under the stack definition if combined with other codes (see below)

BGP when 10 or more of the following codes occur on the same date ("in a stack"), can be across multiple claims

Code	# of genes	Name
81120	1	IDH1 (isocitrate dehydrogenase 1 [NADP+], soluble) (eg, glioma), common variants (eg, R132H, R132C)
81121	1	IDH2 (isocitrate dehydrogenase 2 [NADP+], mitochondrial) (eg, glioma), common variants (eg, R140W, R172M)
81162	2	BRCA1 (BRCA1, DNA repair associated), BRCA2 (BRCA2, DNA repair associated) (eg, hereditary breast and ovarian cancer) gene analysis; full sequence analysis and full duplication/deletion analysis (ie, detection of large gene rearrangements)
81163	2	BRCA1 (BRCA1, DNA repair associated), BRCA2 (BRCA2, DNA repair associated) (eg, hereditary breast and ovarian cancer) gene analysis; familial variants
81164	2	BRCA1 (BRCA1, DNA repair associated), BRCA2 (BRCA2, DNA repair associated) (eg, hereditary breast and ovarian cancer) gene analysis; full duplication/deletion analysis (ie, detection of large gene rearrangements)
81170	1	ABL1 (ABL proto-oncogene 1, non-receptor tyrosine kinase) (eg, acquired imatinib tyrosine kinase inhibitor resistance), gene analysis, variants in the kinase domain
81173	1	AR (androgen receptor) (eg, spinal and bulbar muscular atrophy, Kennedy disease, X chromosome inactivation) gene analysis; full gene sequence

81175	1	ASXL1 (additional sex combs like 1, transcriptional regulator) (eg, myelodysplastic syndrome, myeloproliferative neoplasms, chronic myelomonocytic leukemia), gene analysis; full gene sequence
81191	1	NTRK1 (neurotrophic receptor tyrosine kinase 1) (eg, solid tumors) translocation analysis
81192	1	NTRK2 (neurotrophic receptor tyrosine kinase 2) (eg, solid tumors) translocation analysis
81193	1	NTRK3 (neurotrophic receptor tyrosine kinase 3) (eg, solid tumors) translocation analysis
81194	3	NTRK (neurotrophic receptor tyrosine kinase 1, 2, and 3) (eg, solid tumors) translocation analysis
81201	1	APC (adenomatous polyposis coli) (eg, familial adenomatosis polyposis [FAP], attenuated FAP) gene analysis; full gene sequence
81202	1	APC (adenomatous polyposis coli) (eg, familial adenomatosis polyposis [FAP], attenuated FAP) gene analysis; known familial variants
81203	1	APC (adenomatous polyposis coli) (eg, familial adenomatosis polyposis [FAP], attenuated FAP) gene analysis; duplication/deletion variants
81206	2	BCR/ABL1 (t(9;22)) (eg, chronic myelogenous leukemia) translocation analysis; major breakpoint, qualitative or quantitative
81207	2	BCR/ABL1 (t(9;22)) (eg, chronic myelogenous leukemia) translocation analysis; minor breakpoint, qualitative or quantitative
81208	2	BCR/ABL1 (t(9;22)) (eg, chronic myelogenous leukemia) translocation analysis; other breakpoint, qualitative or quantitative
81210	1	BRAF (B-Raf proto-oncogene, serine/threonine kinase) (eg, colon cancer, melanoma), gene analysis, V600 variant(s)
81212	2	BRCA1 (BRCA1, DNA repair associated), BRCA2 (BRCA2, DNA repair associated) (eg, hereditary breast and ovarian cancer) gene analysis; 185delAG, 5385insC, 6174delT variants
81216	1	BRCA2 (BRCA2, DNA repair associated) (eg, hereditary breast and ovarian cancer) gene analysis; full sequence analysis
81219	1	CALR (calreticulin) (eg, myeloproliferative disorders), gene analysis, common variants in exon 9
81235	1	EGFR (epidermal growth factor receptor) (eg, non-small cell lung cancer) gene analysis, common variants (eg, exon 19 LREA deletion, L858R, T790M, G719A, G719S, L861Q)
81236	1	EZH2 (enhancer of zeste 2 polycomb repressive complex 2 subunit) (eg, myelodysplastic syndrome, myeloproliferative neoplasms) gene analysis, full gene sequence
81242	1	FANCC (Fanconi anemia, complementation group C) (eg, Fanconi anemia, type C) gene analysis, common variant (eg, IVS4+4A>T)
81245	1	FLT3 (fms-related tyrosine kinase 3) (eg, acute myeloid leukemia), gene analysis; internal tandem duplication (ITD) variants (ie, exons 14, 15)
81270	1	JAK2 (Janus kinase 2) (eg, myeloproliferative disorder) gene analysis, p.Val617Phe (V617F) variant
81272	1	KIT (v-kit Hardy-Zuckerman 4 feline sarcoma viral oncogene homolog) (eg, gastrointestinal stromal tumor [GIST], acute myeloid leukemia, melanoma), gene analysis, targeted sequence analysis (eg, exons 8, 11, 13, 17, 18)
81275	1	KRAS (Kirsten rat sarcoma viral oncogene homolog) (eg, carcinoma) gene analysis; variants in exon 2 (eg, codons 12 and 13)
81276	1	KRAS (Kirsten rat sarcoma viral oncogene homolog) (eg, carcinoma) gene analysis; additional variant(s) (eg, codon 61, codon 146)
81279	1	JAK2 (Janus kinase 2) (eg, myeloproliferative disorder) targeted sequence analysis (eg, exons 12 and 13)
81288	1	MLH1 (mutL homolog 1, colon cancer, nonpolyposis type 2) (eg, hereditary non-polyposis colorectal cancer, Lynch syndrome) gene analysis; promoter methylation analysis
81292	1	MLH1 (mutL homolog 1, colon cancer, nonpolyposis type 2) (eg, hereditary non-polyposis colorectal cancer, Lynch syndrome) gene analysis; full sequence analysis
81293	1	MLH1 (mutL homolog 1, colon cancer, nonpolyposis type 2) (eg, hereditary non-polyposis colorectal cancer, Lynch syndrome) gene analysis; known familial variants
81294	1	MLH1 (mutL homolog 1, colon cancer, nonpolyposis type 2) (eg, hereditary non-polyposis colorectal cancer, Lynch syndrome) gene analysis; duplication/deletion variants

© 2026 Chow RD et al. *JAMA Network Open.*

81295	1	MSH2 (mutS homolog 2, colon cancer, nonpolyposis type 1) (eg, hereditary non-polyposis colorectal cancer, Lynch syndrome) gene analysis; full sequence analysis
81296	1	MSH2 (mutS homolog 2, colon cancer, nonpolyposis type 1) (eg, hereditary non-polyposis colorectal cancer, Lynch syndrome) gene analysis; known familial variants
81297	1	MSH2 (mutS homolog 2, colon cancer, nonpolyposis type 1) (eg, hereditary non-polyposis colorectal cancer, Lynch syndrome) gene analysis; duplication/deletion variants
81298	1	MSH6 (mutS homolog 6 [E. coli]) (eg, hereditary non-polyposis colorectal cancer, Lynch syndrome) gene analysis; full sequence analysis
81299	1	MSH6 (mutS homolog 6 [E. coli]) (eg, hereditary non-polyposis colorectal cancer, Lynch syndrome) gene analysis; known familial variants
81300	1	MSH6 (mutS homolog 6 [E. coli]) (eg, hereditary non-polyposis colorectal cancer, Lynch syndrome) gene analysis; duplication/deletion variants
81301	4	Microsatellite instability analysis (eg, hereditary non-polyposis colorectal cancer, Lynch syndrome) of markers for mismatch repair deficiency (eg, BAT25, BAT26), includes comparison of neoplastic and normal tissue, if performed
81307	1	PALB2 (partner and localizer of BRCA2) (eg, breast and pancreatic cancer) gene analysis; full gene sequence
81308	1	PALB2 (partner and localizer of BRCA2) (eg, breast and pancreatic cancer) gene analysis; known familial variant
81309	1	PIK3CA (phosphatidylinositol-4, 5-biphosphate 3-kinase, catalytic subunit alpha) (eg, colorectal and breast cancer) gene analysis, targeted sequence analysis (eg, exons 7, 9, 20)
81310	1	NPM1 (nucleophosmin) (eg, acute myeloid leukemia) gene analysis, exon 12 variants
81311	1	NRAS (neuroblastoma RAS viral [v-ras] oncogene homolog) (eg, colorectal carcinoma), gene analysis, variants in exon 2 (eg, codons 12 and 13) and exon 3 (eg, codon 61)
81314	1	PDGFRA (platelet-derived growth factor receptor, alpha polypeptide) (eg, gastrointestinal stromal tumor [GIST]), gene analysis, targeted sequence analysis (eg, exons 12, 18)
81317	1	PMS2 (postmeiotic segregation increased 2 [S. cerevisiae]) (eg, hereditary non-polyposis colorectal cancer, Lynch syndrome) gene analysis; full sequence analysis
81318	1	PMS2 (postmeiotic segregation increased 2 [S. cerevisiae]) (eg, hereditary non-polyposis colorectal cancer, Lynch syndrome) gene analysis; known familial variants
81319	1	PMS2 (postmeiotic segregation increased 2 [S. cerevisiae]) (eg, hereditary non-polyposis colorectal cancer, Lynch syndrome) gene analysis; duplication/deletion variants
81321	1	PTEN (phosphatase and tensin homolog) (eg, Cowden syndrome, PTEN hamartoma tumor syndrome) gene analysis; full sequence analysis
81323	1	PTEN (phosphatase and tensin homolog) (eg, Cowden syndrome, PTEN hamartoma tumor syndrome) gene analysis; duplication/deletion variant
81338	1	MPL (MPL proto-oncogene, thrombopoietin receptor) (eg, myeloproliferative disorder) gene analysis; common variants (eg, W515A, W515K, W515L, W515R)
81339	1	MPL (MPL proto-oncogene, thrombopoietin receptor) (eg, myeloproliferative disorder) gene analysis; sequence analysis, exon 10
81345	1	TERT (telomerase reverse transcriptase) (eg, thyroid carcinoma, glioblastoma multiforme) gene analysis, targeted sequence analysis (eg, promoter region)
81351	1	TP53 (tumor protein 53) (eg, Li-Fraumeni syndrome) gene analysis; full gene sequence
81400	1	Molecular pathology procedure, Level 1 (eg, identification of single germline variant [eg, SNP] by techniques such as restriction enzyme digestion or melt curve analysis)
81401	1	Molecular pathology procedure, Level 2 (eg, 2-10 SNPs, 1 methylated variant, or 1 somatic variant [typically using nonsequencing target variant analysis], or detection of a dynamic mutation disorder/triplet repeat)
81402	1	Molecular pathology procedure, Level 3 (eg, >10 SNPs, 2-10 methylated variants, or 2-10 somatic variants [typically using non-sequencing target variant analysis], immunoglobulin and T-cell receptor gene rearrangements, duplication/deletion variants of 1 exon, loss of heterozygosity [LOH], uniparental disomy [UPD])
81403	1	Molecular pathology procedure, Level 4 (eg, analysis of single exon by DNA sequence analysis, analysis of >10 amplicons using multiplex PCR in 2 or more independent reactions, mutation scanning or duplication/deletion variants of 2-5 exons)

© 2026 Chow RD et al. *JAMA Network Open.*

81404	1	Molecular pathology procedure, Level 5 (eg, analysis of 2-5 exons by DNA sequence analysis, mutation scanning or duplication/deletion variants of 6-10 exons, or characterization of a dynamic mutation disorder/triplet repeat by Southern blot analysis)
81405	1	Molecular pathology procedure, Level 6 (eg, analysis of 6-10 exons by DNA sequence analysis, mutation scanning or duplication/deletion variants of 11-25 exons, regionally targeted cytogenomic array analysis)
81406	1	Molecular pathology procedure, Level 7 (eg, analysis of 11-25 exons by DNA sequence analysis, mutation scanning or duplication/deletion variants of 26-50 exons)
81407	1	Molecular pathology procedure, Level 8 (eg, analysis of 26-50 exons by DNA sequence analysis, mutation scanning or duplication/deletion variants of >50 exons, sequence analysis of multiple genes on one platform)
81408	1	Molecular pathology procedure, Level 9 (eg, analysis of >50 exons in a single gene by DNA sequence analysis)
81479	unspecified	unlisted molecular pathology procedure. NOTE: this code is counted as a generic code instead if in isolation (see above)

© 2026 Chow RD et al. *JAMA Network Open.*

eTable 3. Regression Results From All Ten Cancer Sites

**Covariate**		**adj. OR**	**P-value**
Medicare type	MA	Reference	
	FFS	1.08 [1.06 - 1.1]	<.001
Medicaid dual eligibility	No	Reference	
	Yes	0.82 [0.8 - 0.85]	<.001
Diagnosis time	2020: Q1	Reference	
	2020: Q2	1.16 [1.1 - 1.22]	<.001
	2020: Q3	1.22 [1.16 - 1.28]	<.001
	2020: Q4	1.29 [1.23 - 1.36]	<.001
	2021: Q1	1.3 [1.24 - 1.36]	<.001
	2021: Q2	1.43 [1.37 - 1.49]	<.001
	2021: Q3	1.65 [1.58 - 1.73]	<.001
	2021: Q4	1.74 [1.66 - 1.81]	<.001
	2022: Q1	1.87 [1.79 - 1.95]	<.001
	2022: Q2	2.11 [2.02 - 2.2]	<.001
Cancer site	Colorectal	Reference	
	Bladder	0.65 [0.61 - 0.69]	<.001
	Breast	0.48 [0.46 - 0.49]	<.001
	Endometrial	0.78 [0.74 - 0.82]	<.001
	Kidney	0.4 [0.37 - 0.42]	<.001
	Lung	2 [1.95 - 2.06]	<.001
	Melanoma	0.86 [0.82 - 0.9]	<.001
	Pancreatic	1.28 [1.22 - 1.33]	<.001
	Prostate	0.36 [0.34 - 0.37]	<.001
	Thyroid	0.53 [0.49 - 0.58]	<.001
Age	66-70	Reference	
	71-75	0.97 [0.95 - 0.99]	.012
	76-80	0.95 [0.93 - 0.98]	<.001
	81+	0.79 [0.76 - 0.81]	<.001
Sex	Male	Reference	
	Female	1.05 [1.03 - 1.08]	<.001
Census region	Northeast	Reference	
	Midwest	0.96 [0.88 - 1.05]	.39
	South	1.13 [1.04 - 1.23]	.005
	West	1.09 [0.99 - 1.2]	.09
	Missing	0.96 [0.78 - 1.19]	.71
Metropolitan residence	Metropolitan	Reference	
	Not-Metropolitan	0.92 [0.9 - 0.95]	<.001
	Missing	1.04 [0.87 - 1.24]	.66
Elixhauser comorbidity	0	Reference	

© 2026 Chow RD et al. *JAMA Network Open.*

	1-2	1.02 [0.99 - 1.04]	.17
	3+	0.91 [0.88 - 0.94]	<.001
CFI frailty	Not Frail	Reference	
	Frail	0.88 [0.86 - 0.91]	<.001
SDI quartile	Quartile 1	Reference	
	Quartile 2	0.98 [0.96 - 1.01]	.23
	Quartile 3	0.94 [0.91 - 0.97]	<.001
	Quartile 4	0.93 [0.9 - 0.95]	<.001
	Missing	0.9 [0.83 - 0.98]	.015
Race and ethnicity	Non-Hispanic White	Reference	
	Asian	1.14 [1.07 - 1.21]	<.001
	Black	1.01 [0.97 - 1.04]	.70
	Hispanic	0.9 [0.86 - 0.94]	<.001
	American Indian/Alaska Native	0.77 [0.64 - 0.92]	.004
	Other	1.08 [0.97 - 1.19]	.15
	Unknown	1.08 [1 - 1.16]	.042

We used the Research Triangle Institute (RTI) codes for race and ethnicity, retaining their original categorization: American Indian/Alaska Native, Asian/Pacific Islander, Hispanic, Black, non-Hispanic White, Other or Unknown.

© 2026 Chow RD et al. *JAMA Network Open.*

eTable 4. Regression Results From Lung Cancers Only

**Covariate**		**adj. OR**	**P-value**
Medicare type	MA	Reference	
	FFS	1.04 [1.01 - 1.08]	.014
Medicaid dual eligibility	No	Reference	
	Yes	0.81 [0.77 - 0.85]	<.001
Diagnosis time	2020: Q1	Reference	
	2020: Q2	1.12 [1.02 - 1.22]	.015
	2020: Q3	1.18 [1.08 - 1.28]	<.001
	2020: Q4	1.29 [1.18 - 1.4]	<.001
	2021: Q1	1.19 [1.11 - 1.28]	<.001
	2021: Q2	1.3 [1.21 - 1.39]	<.001
	2021: Q3	1.61 [1.49 - 1.74]	<.001
	2021: Q4	1.69 [1.57 - 1.82]	<.001
	2022: Q1	1.85 [1.71 - 2]	<.001
	2022: Q2	2 [1.85 - 2.16]	<.001
Age	66-70	Reference	
	71-75	1.04 [1 - 1.09]	.06
	76-80	1.12 [1.06 - 1.17]	<.001
	81+	1.18 [1.12 - 1.24]	<.001
Sex	Male	Reference	
	Female	1.07 [1.03 - 1.11]	<.001
Census region	Northeast	Reference	
	Midwest	0.97 [0.85 - 1.1]	.61
	South	1.07 [0.95 - 1.21]	.28
	West	1.09 [0.94 - 1.26]	.24
	Missing	1.02 [0.72 - 1.45]	.91
Metropolitan residence	Metropolitan	Reference	
	Not-Metropolitan	0.91 [0.86 - 0.95]	<.001
	Missing	1.09 [0.82 - 1.44]	.56
Elixhauser comorbidity	0	Reference	
	1-2	0.91 [0.87 - 0.95]	<.001
	3+	0.76 [0.73 - 0.8]	<.001
CFI frailty	Not Frail	Reference	
	Frail	0.87 [0.83 - 0.91]	<.001
SDI quartile	Quartile 1	Reference	
	Quartile 2	0.96 [0.91 - 1.01]	.10
	Quartile 3	0.89 [0.85 - 0.94]	<.001
	Quartile 4	0.9 [0.85 - 0.94]	<.001
	Missing	0.89 [0.76 - 1.04]	.15
Race and ethnicity	Non-Hispanic White	Reference	

© 2026 Chow RD et al. *JAMA Network Open.*

	Asian	1.55 [1.4 - 1.72]	<.001
	Black	1.1 [1.03 - 1.17]	.004
	Hispanic	0.98 [0.9 - 1.07]	.69
	American Indian/Alaska Native	0.82 [0.62 - 1.08]	.15
	Other	1.36 [1.14 - 1.64]	<.001
	Unknown	1.1 [0.95 - 1.27]	.20

We used the Research Triangle Institute (RTI) codes for race and ethnicity, retaining their original categorization: American Indian/Alaska Native, Asian/Pacific Islander, Hispanic, Black, non-Hispanic White, Other or Unknown.

© 2026 Chow RD et al. *JAMA Network Open.*

eTable 5. Regression Results From Cancers With Explicit BGP Recommendation

Covariate		adj. OR	P-value
Medicare type	MA	Reference	
	FFS	1.04 [1.02 - 1.07]	.002
Medicaid dual eligibility	No	Reference	
	Yes	0.82 [0.79 - 0.85]	<.001
Diagnosis time	2020: Q1	Reference	
	2020: Q2	1.1 [1.03 - 1.17]	.003
	2020: Q3	1.15 [1.08 - 1.22]	<.001
	2020: Q4	1.26 [1.19 - 1.34]	<.001
	2021: Q1	1.18 [1.12 - 1.24]	<.001
	2021: Q2	1.3 [1.23 - 1.37]	<.001
	2021: Q3	1.53 [1.45 - 1.62]	<.001
	2021: Q4	1.6 [1.51 - 1.69]	<.001
	2022: Q1	1.72 [1.62 - 1.81]	<.001
	2022: Q2	1.9 [1.8 - 2.01]	<.001
Cancer site	Lung	Reference	
	Breast	0.24 [0.23 - 0.24]	<.001
	Melanoma	0.43 [0.41 - 0.45]	<.001
	Pancreatic	0.64 [0.61 - 0.66]	<.001
Age	66-70	Reference	
	71-75	0.99 [0.96 - 1.02]	.38
	76-80	0.99 [0.96 - 1.02]	.52
	81+	0.87 [0.84 - 0.9]	<.001
Sex	Male	Reference	
	Female	1.05 [1.02 - 1.08]	.002
Census region	Northeast	Reference	
	Midwest	0.96 [0.87 - 1.07]	.47
	South	1.11 [1.01 - 1.22]	.04
	West	1.07 [0.95 - 1.19]	.28
	Missing	1 [0.78 - 1.29]	.99
Metropolitan residence	Metropolitan	Reference	
	Not-Metropolitan	0.9 [0.87 - 0.94]	<.001
	Missing	1.03 [0.84 - 1.27]	.77
Elixhauser comorbidity	0	Reference	
	1-2	1 [0.97 - 1.03]	.78
	3+	0.87 [0.84 - 0.91]	<.001
CFI frailty	Not Frail	Reference	
	Frail	0.89 [0.86 - 0.92]	<.001
SDI quartile	Quartile 1	Reference	
	Quartile 2	0.99 [0.95 - 1.02]	.46

© 2026 Chow RD et al. *JAMA Network Open.*

	Quartile 3	0.93 [0.9 - 0.97]	<.001
	Quartile 4	0.92 [0.88 - 0.95]	<.001
	Missing	0.89 [0.8 - 0.99]	.028
Race and ethnicity	Non-Hispanic White	Reference	
	Asian	1.26 [1.16 - 1.36]	<.001
	Black	1.02 [0.98 - 1.07]	.33
	Hispanic	0.92 [0.87 - 0.98]	.009
	American Indian/Alaska Native	0.83 [0.67 - 1.04]	.10
	Other	1.04 [0.91 - 1.19]	.54
	Unknown	1.1 [1 - 1.21]	.05

We used the Research Triangle Institute (RTI) codes for race and ethnicity, retaining their original categorization: American Indian/Alaska Native, Asian/Pacific Islander, Hispanic, Black, non-Hispanic White, Other or Unknown.

© 2026 Chow RD et al. *JAMA Network Open.*

eTable 6. Regression Results From Cancers With Equivocal BGP Recommendation

**Covariate**		**adj. OR**	**P-value**
Medicare type	MA	Reference	
	FFS	1.15 [1.11 -1.19]	<.001
Medicaid dual eligibility	No	Reference	
	Yes	0.83 [0.79 - 0.87]	<.001
Diagnosis time	2020: Q1	Reference	
	2020: Q2	1.3 [1.19 - 1.43]	<.001
	2020: Q3	1.39 [1.27 - 1.51]	<.001
	2020: Q4	1.38 [1.27 - 1.51]	<.001
	2021: Q1	1.58 [1.46 - 1.71]	<.001
	2021: Q2	1.71 [1.59 - 1.85]	<.001
	2021: Q3	1.92 [1.78 - 2.08]	<.001
	2021: Q4	2.07 [1.91 - 2.24]	<.001
	2022: Q1	2.23 [2.06 - 2.41]	<.001
	2022: Q2	2.58 [2.39 - 2.79]	<.001
Cancer site	Colorectal	Reference	
	Bladder	0.65 [0.61 - 0.69]	<.001
	Endometrial	0.77 [0.73 - 0.81]	<.001
	Prostate	0.36 [0.34 - 0.37]	<.001
	Thyroid	0.53 [0.48 - 0.57]	<.001
Age	66-70	Reference	
	71-75	0.93 [0.9 - 0.98]	.002
	76-80	0.89 [0.85 - 0.93]	<.001
	81+	0.66 [0.63 - 0.69]	<.001
Sex	Male	Reference	
	Female	1.05 [1.01 - 1.1]	.017
Census region	Northeast	Reference	
	Midwest	0.93 [0.83 - 1.03]	.17
	South	1.16 [1.05 - 1.28]	.004
	West	1.08 [0.96 - 1.21]	.20
	Missing	0.85 [0.62 - 1.16]	.31
Metropolitan residence	Metropolitan	Reference	
	Not-Metropolitan	0.93 [0.89 - 0.97]	.002
	Missing	1.06 [0.83 - 1.36]	.65
Elixhauser comorbidity	0	Reference	
	1-2	1.06 [1.02 - 1.1]	.005
	3+	0.98 [0.94 - 1.03]	.41
CFI frailty	Not Frail	Reference	
	Frail	0.87 [0.83 - 0.91]	<.001
SDI quartile	Quartile 1	Reference	

© 2026 Chow RD et al. *JAMA Network Open.*

	Quartile 2	0.97 [0.93 - 1.02]	.22
	Quartile 3	0.93 [0.89 - 0.98]	.005
	Quartile 4	0.94 [0.89 - 0.99]	.017
	Missing	0.9 [0.79 - 1.03]	.13
Race and ethnicity	Non-Hispanic White	Reference	
	Asian	0.97 [0.88 - 1.08]	.62
	Black	0.99 [0.93 - 1.05]	.64
	Hispanic	0.87 [0.81 - 0.94]	<.001
	American Indian/Alaska Native	0.63 [0.46 - 0.88]	.006
	Other	1.12 [0.96 - 1.32]	.15
	Unknown	1.03 [0.92 - 1.15]	.63

We used the Research Triangle Institute (RTI) codes for race and ethnicity, retaining their original categorization: American Indian/Alaska Native, Asian/Pacific Islander, Hispanic, Black, non-Hispanic White, Other or Unknown.

© 2026 Chow RD et al. *JAMA Network Open.*

eTable 7. Regression Results From Cancers for Which BGP Is Not Routinely Recommended

**Covariate**		**adj. OR**	**P-value**
Medicare type	MA	Reference	
	FFS	1.06 [0.93 - 1.22]	.38
Medicaid dual eligibility	No	Reference	
	Yes	0.79 [0.63 - 0.98]	.036
Diagnosis time	2020: Q1	Reference	
	2020: Q2	1.24 [0.86 - 1.79]	.25
	2020: Q3	1.12 [0.78 - 1.61]	.53
	2020: Q4	1.33 [0.93 - 1.9]	.11
	2021: Q1	1.57 [1.15 - 2.15]	.004
	2021: Q2	1.65 [1.21 - 2.25]	.002
	2021: Q3	1.86 [1.35 - 2.56]	<.001
	2021: Q4	1.75 [1.27 - 2.41]	<.001
	2022: Q1	1.86 [1.36 - 2.55]	<.001
	2022: Q2	2.13 [1.55 - 2.91]	<.001
Age	66-70	Reference	
	71-75	1.06 [0.9 - 1.25]	.51
	76-80	1 [0.84 - 1.2]	.97
	81+	0.84 [0.69 - 1.03]	.10
Sex	Male	Reference	
	Female	1.2 [1.05 - 1.38]	.007
Census region	Northeast	Reference	
	Midwest	0.75 [0.58 - 0.98]	.037
	South	1 [0.79 - 1.28]	.98
	West	1.06 [0.81 - 1.41]	.66
	Missing	0.92 [0.34 - 2.47]	.86
Metropolitan residence	Metropolitan	Reference	
	Not-Metropolitan	0.92 [0.77 - 1.1]	.36
	Missing	0.59 [0.26 - 1.35]	.21
Elixhauser comorbidity	0	Reference	
	1-2	0.99 [0.83 - 1.16]	.86
	3+	0.93 [0.77 - 1.13]	.47
CFI frailty	Not Frail	Reference	
	Frail	0.92 [0.78 - 1.08]	.31
SDI quartile	Quartile 1	Reference	
	Quartile 2	1.07 [0.88 - 1.29]	.49
	Quartile 3	1.16 [0.96 - 1.4]	.13
	Quartile 4	0.95 [0.77 - 1.16]	.59
	Missing	1.14 [0.66 - 1.98]	.64
Race and ethnicity	Non-Hispanic White	Reference	

© 2026 Chow RD et al. *JAMA Network Open.*

	Asian	0.93 [0.59 - 1.49]	.77
	Black	0.84 [0.63 - 1.11]	.22
	Hispanic	0.93 [0.71 - 1.23]	.63
	American Indian/Alaska Native	0.67 [0.2 - 2.24]	.51
	Other	1.08 [0.52 - 2.24]	.84
	Unknown	1.31 [0.87 - 1.97]	.19

We used the Research Triangle Institute (RTI) codes for race and ethnicity, retaining their original categorization: American Indian/Alaska Native, Asian/Pacific Islander, Hispanic, Black, non-Hispanic White, Other or Unknown.

© 2026 Chow RD et al. *JAMA Network Open.*

eMethods.

Addressing potential limitations of MA data

In analyses including Medicare Advantage (MA) beneficiaries, we considered potential limitations of MA encounter data relative to fee-for-service (FFS) claims. Some commonly cited limitations of MA data, such as limited ability to identify associated hospitals or physicians and lack of cost information, were not relevant to this analysis. To minimize potential bias in cohort identification, we excluded diagnosis codes derived from MA chart review records. Chart reviews allow providers to add or delete diagnoses for risk adjustment purposes, which could differentially increase the number of recorded diagnoses among MA beneficiaries. Excluding these records ensured that cancer cohort identification relied on comparable data between MA and FFS beneficiaries and reduced the risk of differential ascertainment.

To ensure completeness of claims/service record capture, we required continuous enrollment in either FFS or MA (without switching) from 12 months before through 6 months after cancer diagnosis. This design provided at least 10 months of observable data prior to potential broad genomic profiling (BGP) testing. We also examined variation in BGP receipt across MA plan types and parent organizations. No outliers were observed by plan type; however, one MA parent organization demonstrated atypical patterns of BGP use and was excluded after review and communication with clinicians at the organization. Finally, we observed a significant interaction between MA enrollment and year of diagnosis with respect to BGP use, suggesting that the observed differences in BGP use were unlikely to be explained by systematic under-ascertainment of testing among MA beneficiaries. Specifically, the divergence in BGP use between FFS and MA emerged over 2020-2022, which would be inconsistent with a fixed, systematic misclassification in MA beneficiaries.

Further discussion on BGP identification

We identified BGP based on CPT codes capturing both tissue-based and liquid biopsy assays. Our algorithm incorporated common billing practices in molecular diagnostics, including "stacking" of multiple biomarker tests billed on the same day. Specifically, patients were classified as receiving BGP if ≥10 biomarkers were billed on the same date, including CPT codes 81400–81408 (molecular pathology procedure, level 1–9), with each level counted as an individual biomarker in the stacked total. We also classified CPT 81445 (genomic sequencing procedures or other molecular multianalyte assays analyzing 5–50 genes) and CPT 81479 (unlisted molecular pathology procedure) as indicating BGP. The full list of codes is in **eTable 2**.

In our prior work, we had implemented a $2000 minimum cost requirement for the generic codes to reduce false positive BGP claims (Wang et al, *JAMA Oncology* 2025). However, this cost threshold could not be implemented for the present study due to the lack of cost information in MA service records in CCW. We assessed the impact of removing the $2000 cost threshold within the FFS cohort for the duration of our study period (where cost information was available), finding that this strategy led to BGP rates that correlated well with our previously described approach.

We did not attempt to distinguish focused or single-gene testing (eg, hotspot panels or individual biomarker assays) from no molecular testing. Several clinically relevant biomarkers lack unique CPT codes (eg, ALK), and common laboratory methods used to detect biomarkers, such as immunohistochemistry or fluorescence in situ hybridization, are also used for other diagnostic purposes despite having CPT codes. Therefore, patients receiving such testing were conservatively classified within the non-BGP category.

© 2026 Chow RD et al. *JAMA Network Open.*
