# Supplementary material for: Medicare Insurance Type and Broad Genomic Profiling in Metastatic Cancer
Source: JAMA Netw Open. 2026 May 27;9(5):e2614919. doi: 10.1001/jamanetworkopen.2026.14919 (PMC13216988; doi:10.1001/jamanetworkopen.2026.14919)
Supplement: Supplement 2. — Data Sharing Statement [file jamanetwopen-e2614919-s002.pdf]

Data Sharing Statement

Chow. Medicare Insurance Type and Broad Genomic Profiling in Metastatic Cancer. *JAMA Netw Open*. Published May 27, 2026. doi:10.1001/jamanetworkopen.2026.14919

Data
Data available: No

Additional Information
Explanation for why data not available: The data underlying this article were accessed via the Virtual Research Data Center with permission under a data use agreement and cannot be shared. Centers for Medicare and Medicaid Services Research Identifiable Files data are available to investigators for research purposes and can be requested through the Research Data Assistance Center at https://resdac.org/.
